# Proteomics reveals periodontitis-driven oxidative stress and lipid metabolism disruption in NAFLD

**DOI:** 10.3389/fendo.2025.1600015

**Published:** 2025-08-28

**Authors:** Boyuan Sun, Xiaomeng Liu, Yixuan Jiang, ShuangYan Qi, Zhengyu Guan, Hongjiao Li

**Affiliations:** Department of Stomatology, Xinhua Hospital Affiliated to Shanghai Jiao Tong University School of Medicine, Shanghai, China

**Keywords:** periodontitis, NAFLD, oxidative stress, lipid metabolism, proteomics

## Abstract

**Background:**

Periodontitis is increasingly recognized as a risk factor for non-alcoholic fatty liver disease (NAFLD), yet the underlying mechanisms remain unclear. Oxidative stress and lipid metabolism dysregulation may serve as key pathological links. This study investigates the impact of periodontitis on NAFLD progression and identifies molecular targets involved in hepatic oxidative stress and lipid alterations.

**Materials and methods:**

A rat model of periodontitis was established via molar ligation. Hepatic pathology was assessed using histological staining, biochemical assays, and oxidative stress markers. Proteomic analysis identified differentially expressed proteins (DEPs) associated with lipid metabolism and inflammation. Functional enrichment analysis, protein-protein interaction (PPI) network construction, and hub proteins identification were conducted.

**Results:**

Periodontitis significantly exacerbated hepatic lipid accumulation, fibrosis, and oxidative stress. Proteomic analysis identified 244 DEPs enriched in metabolic and inflammatory pathways. PPI network analysis revealed ACOX1, DBT, ACAA2, and HADHA as hub proteins. Downregulation of these proteins correlated with impaired lipid oxidation and hepatocellular injury in periodontitis-induced NAFLD.

**Conclusion:**

Periodontitis accelerates NAFLD progression through oxidative stress and lipid metabolism dysfunction. ACOX1, DBT, ACAA2, and HADHA may serve as therapeutic targets. These findings highlight the importance of oral health in systemic metabolic disorders and suggest new intervention strategies.

## 1 Introduction

Non-alcoholic fatty liver disease (NAFLD) is a severe condition with a global prevalence of approximately 25.2% (1). It exhibits a progressive course, encompassing a spectrum of liver disorders with varying degrees of damage and fibrosis (2). Simple steatosis is classified as NAFL, while non-alcoholic steatohepatitis (NASH) represents a more severe stage characterized by inflammation and hepatocellular injury. Recently, NAFLD has been redefined as MAFLD to better emphasize its association with metabolic dysfunction. In this study, the term NAFLD is retained for consistency with existing literature; however, our findings are also consistent with the diagnostic criteria and pathophysiological features of MAFLD. Key pathological features include steatosis, hepatocyte ballooning, and lobular inflammation with varying degrees of fibrosis (3). Currently, treatment options for NAFLD remain limited, underscoring the urgent need to identify novel therapeutic targets.

Increasing evidence suggests that NAFLD is a systemic disorder influenced by lipid metabolism dysfunction (4). Excessive lipolysis, fructose-driven lipogenesis, impaired lipid oxidation, and the accumulation of lipotoxic species contribute to hepatic lipid overload and metabolic dysregulation (5–8). Additionally, oxidative stress has garnered significant attention in NAFLD pathogenesis. It not only promotes disease progression by disrupting lipid metabolism but also directly induces hepatocellular injury, exacerbating apoptosis and inflammation (9). Studies have consistently reported oxidative stress in the livers of NAFLD patients (10, 11), with reactive oxygen species (ROS) accumulation further aggravating lipid metabolism abnormalities, creating a vicious cycle (12, 13). Notably, randomized clinical trials have demonstrated that antioxidant therapy, such as vitamin E, significantly improves NASH progression (14).

Periodontitis is a chronic progressive inflammatory disease and ranks as the sixth most prevalent condition worldwide. Recent epidemiological studies indicate that approximately 42.2% of the U.S. population is affected, with an even higher prevalence of 90% in China (15). Severe periodontitis not only compromises oral health but is also closely linked to systemic diseases such as type 2 diabetes, Alzheimer’s disease, and rheumatoid arthritis (16). Epidemiological evidence suggests that individuals with moderate to severe periodontitis are at a higher risk of developing NAFLD (17). Periodontitis may contribute to NAFLD by increasing systemic inflammatory burden, thereby disrupting metabolic and immune homeostasis (18). Notably, periodontal treatment has been shown to reduce serum levels of aspartate aminotransferase (AST) and alanine aminotransferase (ALT) in patients with NAFLD/NASH (19). Furthermore, in a ligature-induced rat model of experimental periodontitis, the disease was found to promote hepatic steatosis by exacerbating oxidative stress and lipid peroxidation, suggesting that oxidative stress may play a key role in periodontitis-induced NAFLD (20). Investigating oxidative stress and lipid metabolism alterations in the context of periodontitis and NAFLD comorbidity may offer novel therapeutic insights for NAFLD treatment.

Recent insights into the pathogenesis of NAFLD have underscored the significance of inter-organ communication, particularly the gut-liver axis (21). However, increasing attention is being paid to the “oral-liver axis,” a conceptual framework that highlights the impact of oral pathogens and inflammatory mediators on liver function (16, 22). The oral cavity, especially in the context of periodontitis, serves as a reservoir of pathogenic bacteria and pro-inflammatory cytokines that can enter systemic circulation and influence distant organs, including the liver (23). Multiple animal studies have demonstrated that oral administration of *P. gingivalis* and *A. actinomycetemcomitans* induces gut microbiota alterations and triggers insulin resistance along with hepatic lipid deposition through disruption of glucose and lipid metabolic pathways (24, 25). Beyond bacterial pathogens, the characteristic systemic low-grade inflammatory state of periodontitis elevates circulating levels of C-reactive protein, IL-6 and TNF-α (26, 27), representing another crucial pathway linking periodontitis to NAFLD. TNF-α plays a pivotal role in hepatic insulin resistance by activating serine/threonine kinases that phosphorylate and inactivate insulin receptor substrates, thereby blocking insulin receptor signaling cascades (28). Interleukin-6, upregulated by TNF-α, is similarly associated with impaired insulin signaling, induced fatty acid oxidation, and hepatic C-reactive protein production (29, 30). Given the potential driving role of periodontitis in NAFLD pathogenesis, elucidating the molecular mechanisms underlying this oral-liver interaction—particularly the regulatory networks involving oxidative stress and lipid metabolism—is critical for developing novel intervention strategies against NAFLD.

This study aims to investigate the causal relationship between periodontitis and NAFLD and to elucidate the underlying mechanisms involved. An experimental rat model of periodontitis was established using molar ligation, and systemic and hepatic pathological markers were assessed to evaluate oxidative stress and lipid accumulation in the liver. Comprehensive mass spectrometry-based proteomic analysis was then conducted to identify key lipid metabolic pathways and protein targets involved in the pathological progression of NAFLD induced by periodontitis.

## 2 Materials and methods

### 2.1 Materials

Micro-CT system (SCANC, Switzerland); Oil Red O staining kit (Solarbio, Beijing, China); biochemical assay kits for superoxide dismutase (SOD), glutathione (GSH), malondialdehyde (MDA), alanine aminotransferase (ALT), and aspartate aminotransferase (AST) (Nanjing Jiancheng Bioengineering Institute, China); reverse transcription kit (TaKaRa, Japan); primers synthesized by Sangon Biotech (Shanghai, China); antibodies for JNK, phosphorylated c-Jun N-terminal kinase (P-JNK), NF-κB, and Caspase-3 (Cell Signaling Technology, USA); secondary antibodies (Proteintech, USA); paraffin microtome (Thermo, USA); real-time quantitative PCR system (Agilent, USA); TUNEL assay kit; electrochemiluminescence (ECL) detection system; optical and fluorescence microscopes (Olympus, Japan).

### 2.2 Establishment of the periodontitis rat model

Twelve specific pathogen-free (SPF) male Wistar rats (6 weeks old, 200–220 g) were randomly divided into a control group (n = 6) and a periodontitis group (n = 6). The induction of periodontitis in rats was performed as previously described (31–33). All rats were obtained from the Experimental Animal Center of Shanghai Jiao Tong University. In the periodontitis group, rats were anesthetized with an intraperitoneal injection of 2% pentobarbital sodium, and a 0.2 mm orthodontic ligature wire was placed around the cervical region of the maxillary first molars bilaterally to induce periodontitis. After eight weeks, periodontal clinical parameters were assessed, and the rats were euthanized. Maxillary bone, liver, and blood samples were immediately collected for further analysis. All animal experiments were approved by the Ethics Committee of Xinhua Hospital, Shanghai Jiao Tong University School of Medicine (XHEC-NSFC-2019-240) and conducted following ARRIVE guidelines. Rats were housed under pathogen-free conditions at 22°C–26°C with a humidity of 40%–60%.

### 2.3 Micro-CT analysis

The maxillary bone was scanned using a Micro-CT system with the following parameters: 70 kV, 200 mA, 300 ms exposure time, voxel size 10 μm × 10 μm × 10 μm. Image processing and 3D reconstruction of the alveolar bone were performed using IPL software. ImageJ was used to measure the distance from the cementoenamel junction (CEJ) to the alveolar crest at the mesiobuccal, midbuccal, and distobuccal sites of the maxillary first molars.

### 2.4 Hematoxylin-eosin staining

Liver tissues were fixed in 4% paraformaldehyde, embedded in paraffin, and sectioned. Standard HE staining was performed for histopathological examination under a microscope.

### 2.5 Oil red O staining

Frozen liver sections were stained using an Oil Red O staining kit to evaluate hepatic steatosis. The sections were fixed in 4% paraformaldehyde, washed, and incubated with Oil Red O solution for 10 minutes, followed by 60% isopropanol differentiation. Hematoxylin counterstaining was performed for 5 minutes, and the slides were sealed with glycerol gelatin. Images were captured under a light microscope, and hepatic lipid accumulation was quantified using Image-Pro Plus 6.0.

### 2.6 Liver function and oxidative stress assays

Serum ALT and AST levels and hepatic oxidative stress markers SOD, MDA, and GSH were measured using commercial biochemical assay kits, following the manufacturer’s instructions.

### 2.7 TUNEL staining

Paraffin-embedded liver sections were stained using a TUNEL assay kit (34), followed by Hoechst staining for nuclear visualization. Apoptotic cells were observed under a fluorescence microscope.

### 2.8 Statistical analysis

All statistical analyses were performed using GraphPad Prism 6.0. Data were expressed as mean ± standard deviation ( $\bar{\text{x}}$ ± s). Group comparisons were conducted using the t-test.

### 2.9 Protein identification

A stringent data filtering approach was applied: the false discovery rate (FDR) for peptide and protein identification was set at 1%, and proteins were required to contain at least one unique peptide for validation.

### 2.10 Protein extraction

Three rats were randomly selected from both the periodontitis and control groups. Gingival tissues were collected, labeled with sample group and collection date, and stored in liquid nitrogen. Samples were lysed in SDT lysis buffer, homogenized twice using an MP homogenizer (24×2, 6.0 M/S, 60 s), subjected to ultrasonic homogenization, and incubated in a boiling water bath for 10 minutes. After centrifugation (14,000 rpm, 15 min), the supernatant was filtered through a 0.22 µm centrifuge tube, and total protein was quantified using the BCA assay. Samples were aliquoted and stored at −80°C.

### 2.11 SDS-PAGE separation

Protein samples were mixed with 6× loading buffer, boiled for 5 minutes, and separated using 12% SDS-PAGE. Proteins were visualized with Coomassie Brilliant Blue R-250 staining.

### 2.12 FASP digestion

50–200 μg of protein per sample was reduced in 100 mM DTT at 100°C for 5 minutes. Proteins were processed using a 30 kDa ultrafiltration unit with 8 M urea + 150 mM Tris-HCl (pH 8.5) buffer and centrifuged at 12,500 rpm for 15 minutes, with repeated washes (35). 100 µL IAA buffer was added and incubated for 30 minutes at room temperature in the dark. The sample was washed twice with UA buffer, followed by 50 mM NH4HCO3 buffer, before replacing the ultrafiltration tube. Proteins were digested overnight at 37°C with 40 µL Trypsin buffer (4 µg Trypsin in 40 µL 50 mM NH4HCO3). Peptides were collected, desalted using a C18 cartridge, and freeze-dried before resuspension in 0.1% formic acid (40 µL).

### 2.13 Mass spectrometry analysis

Peptide separation was performed using a nano-flow Easy-nLC system at a flow rate of 300 nL/min. Samples were analyzed using an Orbitrap Exploris 480 mass spectrometer for 90 minutes.

### 2.14 Data processing

MS data were analyzed using SpectroMine software (version 2.6.210114.47784, Biognosys AG). Peptide identification was performed against the Uniprot_HomoSapiens_20394_20210127 database, with an initial precursor mass tolerance of 6 ppm. The search parameters followed trypsin/P cleavage rules, with carbamidomethylation of cysteine as a fixed modification and N-terminal acetylation and methionine oxidation as variable modifications. The global false discovery rate (FDR) was set at 0.01. Protein abundance was calculated based on normalized spectral intensity, and proteins with fold change > 2 or < 0.5 and p < 0.05 (Student’s t-test) were considered differentially expressed (36).

### 2.15 Clustering analysis

Normalized quantitative data of target proteins were analyzed using matplotlib software to calculate sample distances and expression levels (distance metric: Euclidean; linkage method: Average linkage), generating hierarchical clustering heatmaps.

### 2.16 Enrichment analysis

Functional enrichment analysis of differentially expressed proteins was conducted using the “clusterProfiler” R package (v4.0). This included Gene Ontology (GO) and Kyoto Encyclopedia of Genes and Genomes (KEGG) pathway analyses to identify biological processes, molecular functions, and pathways related to lipid metabolism and oxidative stress. The most representative terms were selected based on keyword similarity, with statistical significance set at p < 0.05.

### 2.17 Gene set enrichment analysis

GSEA was performed using Gene Set Enrichment Analysis software (Broad Institute). The dataset was derived from the msigdbr package (V7.5.1) in R for KEGG pathway analysis (37).

### 2.18 PPI network and hub proteins identification

PPI network prediction was performed using the STRING database (combined interaction score > 0.4) with one intermediate protein allowed to be added to connect proteins and visualization was carried out with Cytoscape software (38). Hub proteins were identified based on the topological structure of the PPI network, using the cytoHubba plugin to recognize key targets or subnetworks. The algorithms used include: BottleNeck, MCC, EPC, Stress, Radiality, Degree, DMNC, Closeness, and MNC (39).

### 2.19 Statistical analysis

All statistical analyses were conducted using R software (version 3.3.1). Protein quantification reproducibility was assessed using Pearson’s correlation coefficient and principal component analysis (PCA). A p-value < 0.05 was considered statistically significant for differentially expressed proteins between groups.

## 3 Result

### 3.1 Pathological changes in the liver tissue of periodontitis-induced rats compared to healthy controls

**Figure 1:**
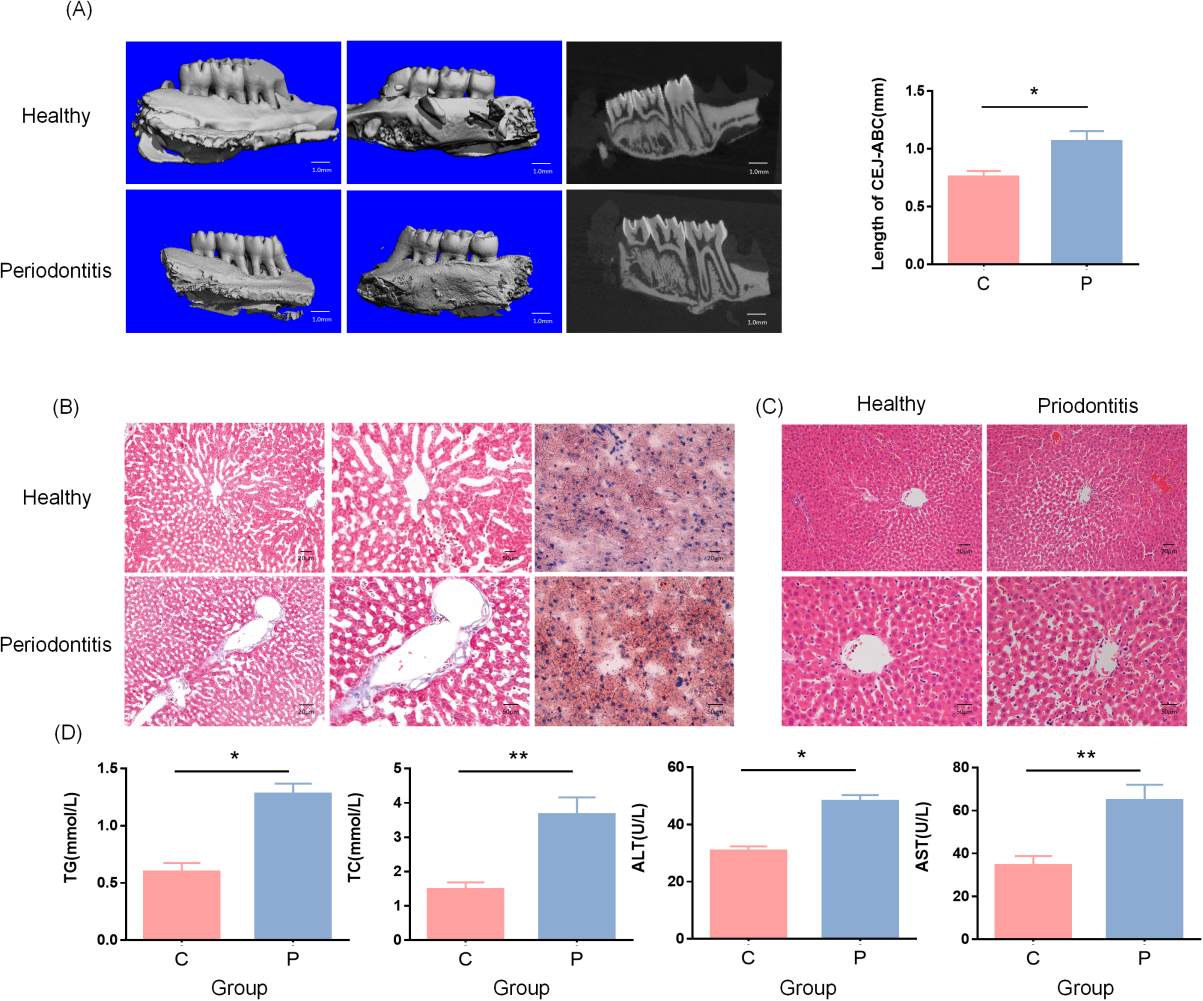
Periodontitis exacerbates NAFLD progression. **(A)** Micro-CT analysis (Scale bar = 1.0 mm). The distance from the cementoenamel junction (CEJ) to the alveolar crest was measured and presented as mean ± standard deviation. *p < 0.05. **(B)** Compared to the control group, the periodontitis group showed significant increases in serum TG, TC, ALT, and AST. *p < 0.05, **p < 0.005. **(C)** Oil Red O and Masson staining. The periodontitis group exhibited increased hepatic lipid droplet accumulation and fibrosis compared to the control group. **(D)** HE staining. Hepatic tissue from the periodontitis group showed disrupted hepatic cord arrangement and lipid droplet accumulation compared to controls. Yellow arrows indicate lipid droplets. C represents the control group, and P represents the periodontitis group.

To investigate the impact of periodontitis on the progression of NAFLD in rats, we established a periodontitis model (PD) by ligating the maxillary second molars bilaterally with silk sutures and applying subgingival plaque from periodontitis patients for eight weeks. As expected, periodontitis resulted in significant alveolar bone resorption (**Figure 1A**). To assess liver function in periodontitis-induced rats, biochemical analysis was performed on blood samples. The results showed a significant increase in ALT, AST and ALP levels (p < 0.05), indicating hepatocellular injury and impaired liver function. Additionally, total cholesterol (TC) and triglyceride (TG) levels were significantly elevated (p < 0.05), suggesting lipid metabolism disorders, which may further contribute to hepatic fat accumulation (**Figure 1B**). Histopathological analysis was conducted on liver tissue sections to evaluate liver damage. Hematoxylin and eosin (HE) staining of liver tissue revealed disorganized hepatic cord arrangement in the periodontitis group, with inflammatory cell infiltration observed in both the hepatic lobules and portal areas. White lipid vacuoles were present in the cytoplasm of hepatocytes (**Figure 1C**). Oil Red O staining showed that the red lipid droplets in the cytoplasm of liver tissue were significantly more abundant in the periodontitis group than in the control group, while Masson’s trichrome staining demonstrated increased fibrosis compared to control rats (**Figure 1D**). Collectively, these findings indicate that periodontitis leads to liver dysfunction and pathological alterations in rats, supporting a potential link between periodontitis and NAFLD progression.

### 3.2 Periodontitis induces significant oxidative stress in rat liver

**Figure 2:**
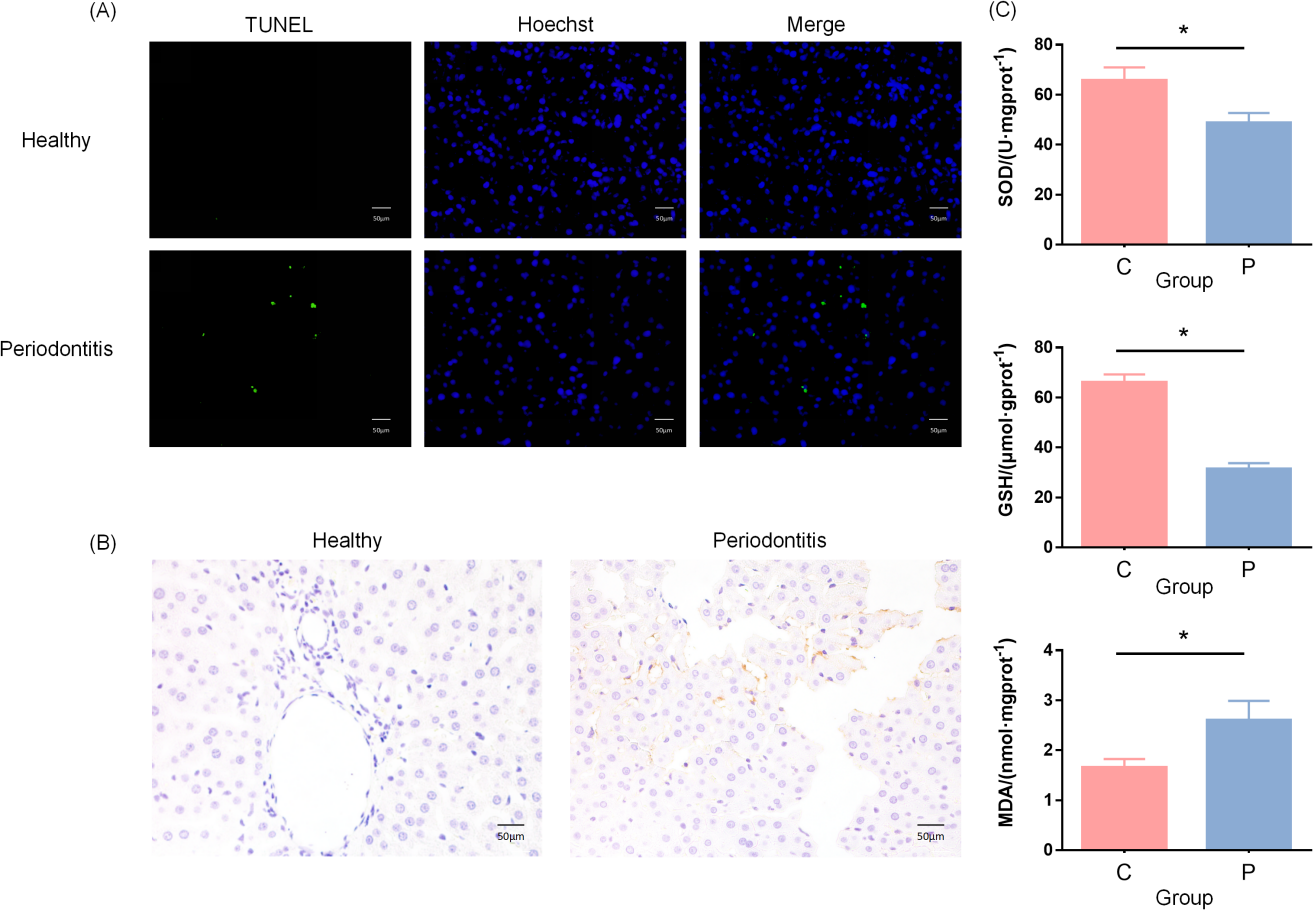
Oxidative stress in the liver of periodontitis-induced rats. **(A)** Representative images showing TUNEL-positive apoptotic cells (green) and Hoechst (blue) staining in hepatic tissues of periodontitis mice and healthy mice. The merged images illustrate the co-localization of apoptotic cells and nuclei. **(B)** Immunohistochemistry staining showed significant NF-kB activation in hepatocytes of periodontitis-induced rats. **(C)** Serum analysis revealed decreased GSH and SOD levels and elevated MDA levels, indicating enhanced oxidative stress. *p < 0.05.

To investigate the mechanisms underlying liver pathology in periodontitis-induced rats, we assessed hepatocyte apoptosis using TUNEL staining. Compared to the control group, the periodontitis group exhibited a significant increase in TUNEL-positive cells, suggesting that systemic inflammation triggered by periodontitis may contribute to hepatocyte apoptosis (**Figure 2A**). Immunohistochemical analysis of NF-κB pathway activation revealed a marked upregulation of NF-κB expression in the livers of periodontitis-induced rats, particularly in central and periportal regions, where staining intensity was notably enhanced. This suggests that periodontitis may activate the NF-κB signaling pathway, promoting the release of inflammatory mediators, exacerbating oxidative stress, and further driving hepatocyte damage and apoptosis (**Figure 2B**). Additionally, biochemical analysis showed that serum superoxide dismutase (SOD) and glutathione (GSH) levels were significantly lower in periodontitis-induced rats compared to controls, while malondialdehyde (MDA) levels were elevated. These findings indicate a diminished antioxidant capacity and a pronounced oxidative stress state in periodontitis-induced rats (**Figure 2C**).

### 3.3 Protein expression in the livers of periodontitis-induced rats

**Figure 3:**
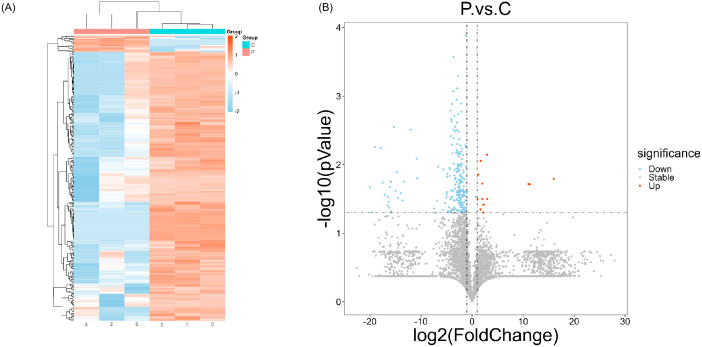
Significant differences in proteomic characterization between normal and PD rats. **(A)** The clustering of differentially expressed proteins is represented by the heat map. Each column represents a sample, and different groups are distinguished by long bars of different colors, with the normal rats (C group) represented by blue and the periodontitis-induced rats (P group) by orange; each row represents a differential protein; and the relative content of differential proteins is indicated by differences in color. **(B)** Volcano plot was drawn using two factors, the fold change (FC) of protein expression differences between the gingival tissue of young mice and old mice and the p-value together. Blue circles on the left side of the graph represent the down-regulation of expression, and orange circles on the right side represent the up-regulation of expression (FC ≥ 1.2, p ≤ 0.05).

Building on our initial findings, we further analyzed protein expression changes in the liver tissues of periodontitis-induced rats to explore the molecular mechanisms underlying disease pathology. Liver samples from both the periodontitis and control groups were subjected to proteomic analysis using 4D label-free technology, with three randomly selected samples from each group for comparison. A total of 5,526 proteins were identified across both groups. Hierarchical clustering heatmaps revealed distinct differences in protein expression between periodontitis-induced and control rats (**Figure 3A**). Based on a fold change (FC) > 1.2 and p < 0.05, 244 differentially expressed proteins were identified, with 14 proteins upregulated and 230 proteins downregulated in the periodontitis group (**Figure 3B**).

### 3.4 Differentially expressed proteins are primarily associated with oxidative stress, inflammation, and metabolic processes

**Figure 4:**
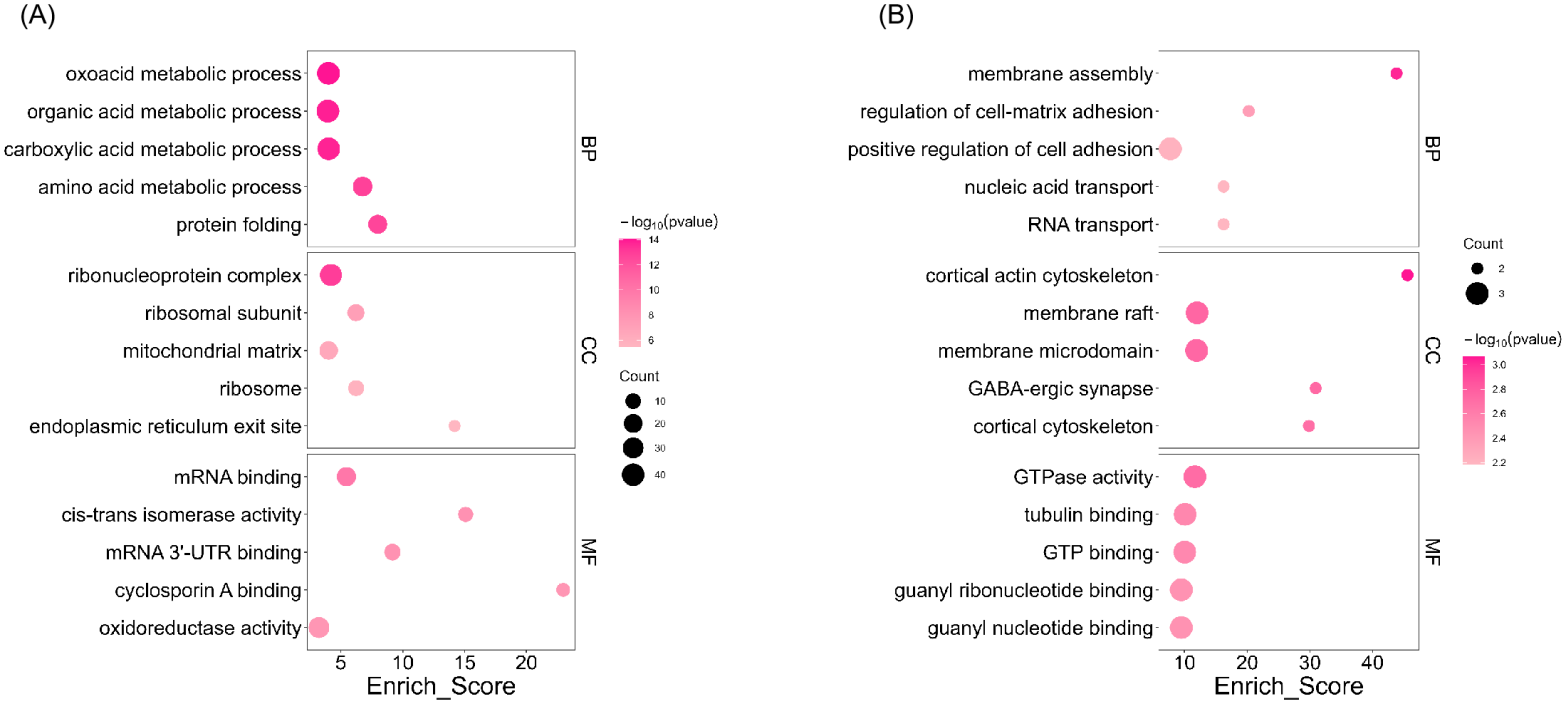
Gene ontology (GO) enrichment analysis based on DEPs. **(A)** The top 10 most enriched GO terms in biological process (BP), cellular component (CC), and molecular function (MF) for downregulated proteins in the liver of periodontitis-induced rats. **(B)** The top 10 most enriched GO terms in BP, CC, and MF for upregulated proteins in the gingival tissue of periodontitis-induced rats. Dot size corresponds to the number of enriched proteins (Count), color intensity represents the statistical significance as measured by -log_10_(p-value), and the x-axis indicates the enrichment score.

**Figure 5:**
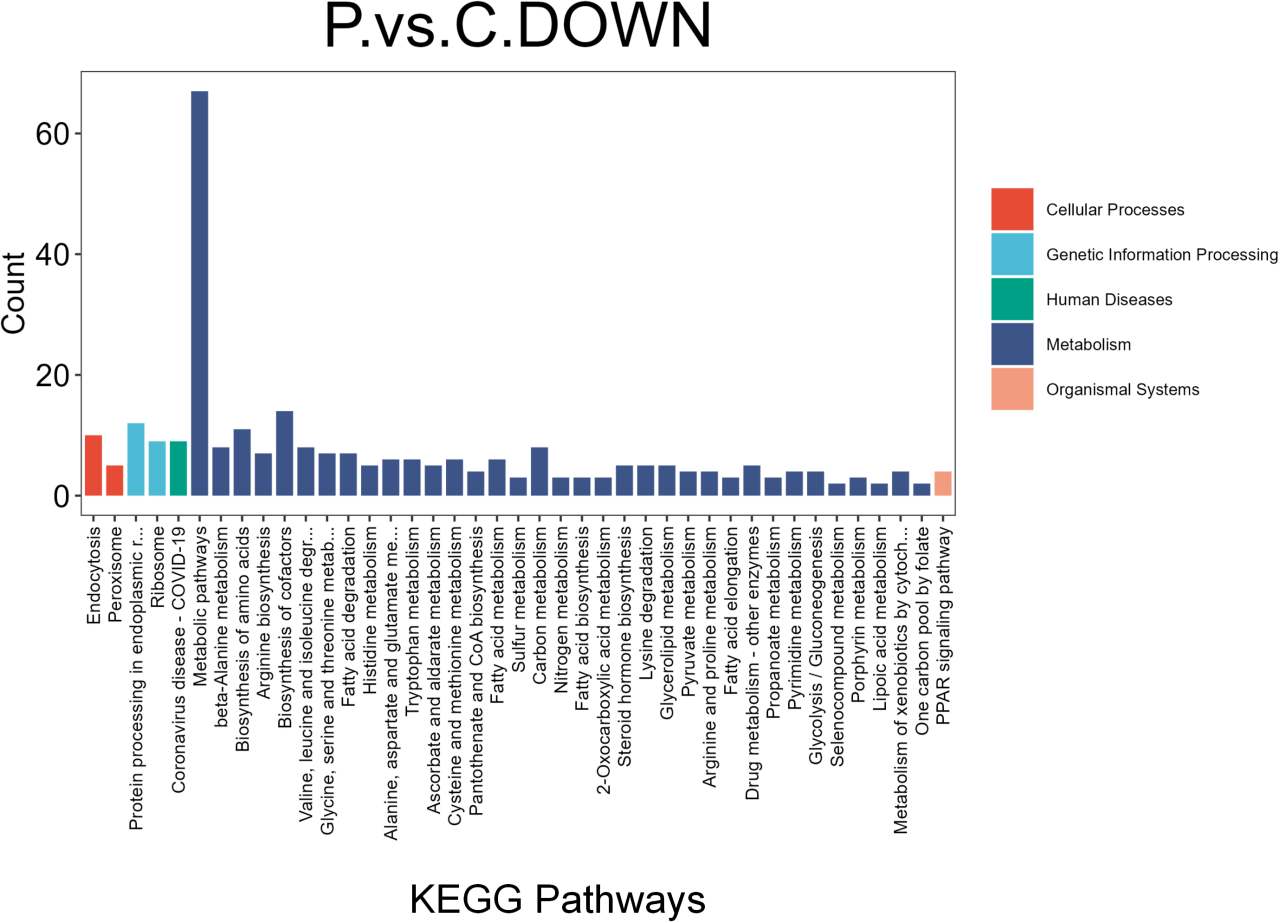
Kyoto encyclopedia of genes and genomes (KEGG) enrichment analysis based on down regulated DEPs. Bar plot of KEGG enrichment analysis outcomes. The DEPs are primarily enriched in metabolism-related pathways.

**Figure 6:**
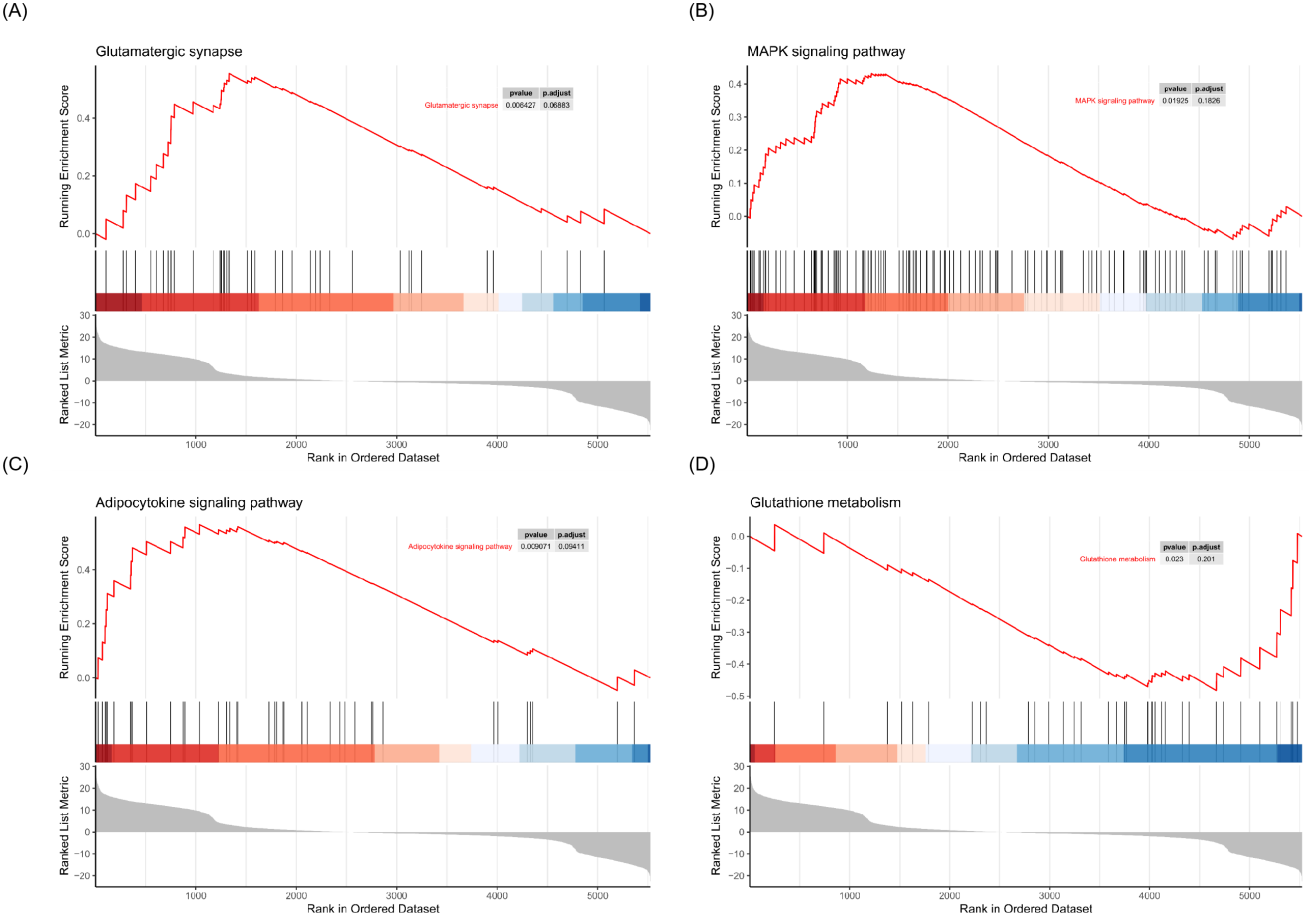
Gene set enrichment analysis (GSEA). **(A)** GSEA results for the Glutamatergic synapse pathway. **(B)** GSEA results for the MAPK signaling pathway. **(C)** GSEA results for the Adipocytokine signaling pathway. **(D)** GSEA results for Glutathione metabolism.

To determine the biological processes associated with the 244 differentially expressed proteins, we performed Gene Ontology (GO) and Kyoto Encyclopedia of Genes and Genomes (KEGG) enrichment analyses. GO enrichment analysis revealed that downregulated proteins in the livers of periodontitis-induced rats were mainly involved in metabolic processes, including oxoacid, organic acid, carboxylic acid, and amino acid metabolism (BP), as well as molecular functions related to oxidoreductase activity and cis-trans isomerase activity (MF) (**Figure 4A**). In contrast, upregulated proteins were enriched in biological processes related to membrane assembly, regulation of cell-matrix adhesion, and positive regulation of cell adhesion (BP), as well as molecular functions such as GTPase activity, tubulin binding, and GTP binding (MF) (**Figure 4B**). KEGG enrichment analysis indicated that downregulated proteins were primarily associated with metabolic pathways, including amino acid metabolism (arginine biosynthesis, histidine metabolism, valine/leucine/isoleucine degradation, lysine degradation, and arginine/proline metabolism), lipid metabolism (fatty acid biosynthesis and degradation), and carbohydrate metabolism (glycolysis/gluconeogenesis and one-carbon metabolism via folate). Additionally, differentially expressed proteins were enriched in the PPAR signaling pathway (**Figure 5**). Gene Set Enrichment Analysis (GSEA) further revealed that the MAPK signaling pathway (NES = 1.37, p < 0.05), glutamatergic synapse (NES = 1.54, p < 0.05), and adipocytokine signaling pathway (NES = 1.54, p < 0.05) were upregulated in periodontitis-induced rats compared to controls, while glutathione metabolism (NES = -1.46, p < 0.05) was downregulated (**Figure 6**). Collectively, these findings indicate that differentially expressed proteins in the livers of periodontitis-induced rats are predominantly linked to oxidative stress, inflammation, and metabolic dysregulation.

### 3.5 Identification of differentially expressed proteins related to lipid metabolism, construction of PPI network, and screening of core proteins

**Figure 7:**
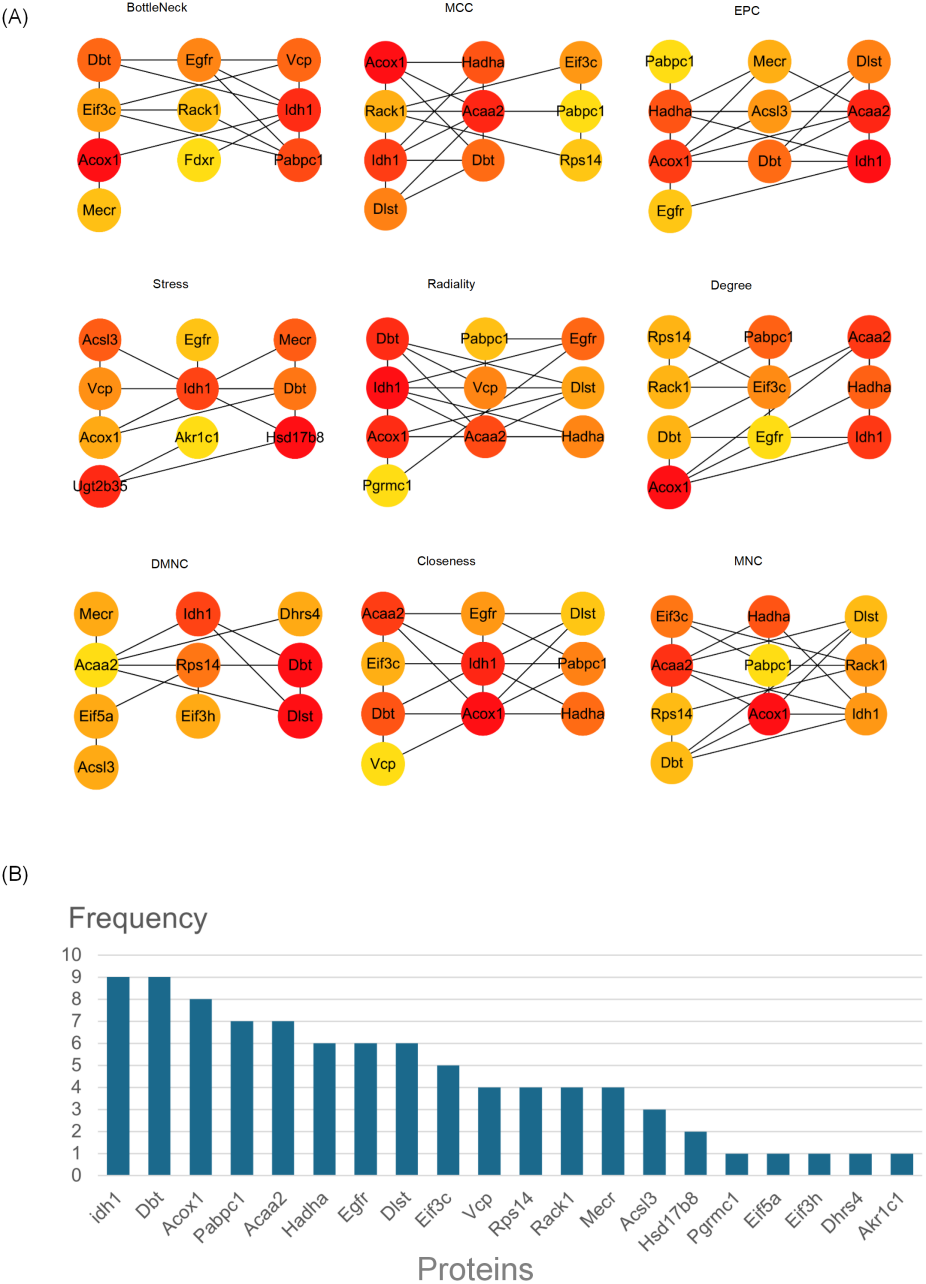
PPI network identified hub proteins. **(A)** Top 10 hub proteins identified using the cytoHubba plugin in Cytoscape, based on nine different algorithms, BottleNeck, MCC, EPC, Stress, Radiality, Degree, DMNC, Closeness and MNC. **(B)** Frequency of hub proteins occurrence across the nine algorithms.

Based on the aforementioned results, we hypothesized that the hepatic pathological changes induced by periodontitis are closely associated with alterations in hepatic metabolic processes. GO and KEGG analyses revealed that the differentially expressed proteins (DEPs) were significantly enriched in lipid metabolism-related pathways, suggesting that changes in lipid metabolism pathways may play a pivotal role in the hepatic pathological changes triggered by periodontitis. We manually screened DEPs enriched in GO and KEGG lipid metabolism-related entries and supplemented them using the Rat Genome Database (RGD), ultimately identifying 56 lipid metabolism-related DEPs (LRPs). These LRPs were uploaded to the STRING database for processing, and a Protein-Protein Interaction (PPI) network was constructed using Cytoscape software. The resulting network comprised 43 nodes and 136 edges, with combined scores > 0.4 (**Figure 7A**). Subsequently, we employed Cytoscape’s plugin CytoHubba and utilized nine different algorithms—bottleneck, closeness, degree, DMNC, EPC, MCC, MNC, radiality, and stress—to calculate the top 10 hub proteins. The frequency of these proteins is illustrated in the figure. The top-ranked proteins were IDH1, DBT, ACOX1, PABPC1, ACAA2, HADHA, EGFR, DLST, EIF3C, and VCP (**Figure 7B**). In summary, these proteins are likely to be core lipid metabolism-related proteins (LRPs) involved in the hepatic pathological changes associated with periodontitis.

## 4 Discussion

Periodontitis has been shown to disrupt lipid metabolism, leading to systemic damage across multiple organs, including blood vessels (40), the heart (41), kidneys (42), and brain (43). Studies suggest that lipid metabolism disorders can cause hepatocyte injury through abnormal lipid accumulation, which is closely linked to the progression of NAFLD (44). Despite these associations, direct evidence linking periodontitis-induced lipid metabolism dysfunction to NAFLD progression remains limited. In this study, we established a combined rat model of periodontitis and NAFLD and, for the first time, demonstrated that periodontitis exacerbates NAFLD progression by inducing oxidative stress and disrupting hepatic lipid metabolism. Specifically, periodontitis triggers oxidative stress, leading to lipid metabolic dysregulation, abnormal hepatic lipid accumulation, and accelerated NAFLD development.

Periodontitis Accelerates the Progression of NAFLD. Our data clearly demonstrate that periodontitis exacerbates NAFLD progression. In this study, we focused on the role of periodontitis, a disease closely linked to lipid metabolism, in the pathogenesis of NAFLD. We observed significant liver dysfunction and hepatocyte damage in periodontitis-induced rats. NAFLD is a metabolic liver disease characterized by excessive lipid accumulation in hepatocytes, leading to cellular injury, hepatic stellate cell activation, and inflammatory responses, ultimately driving chronic liver inflammation and fibrosis (2). Interestingly, epidemiological studies have shown that periodontitis patients exhibit lipid accumulation in multiple organs, while periodontal treatment can improve surrogate markers of lipid abnormalities, such as total cholesterol (TC) (45–48) and triglycerides (TG) (45, 47–49). Additionally, clinical evidence suggests that periodontitis is associated with impaired liver function, whereas intensive periodontal treatment significantly improves liver function (19). Despite reports of the detrimental effects of periodontitis on liver function, direct evidence linking periodontitis to NAFLD remains limited. Our study demonstrates that periodontitis significantly worsens hepatic lipid metabolism disorders, indicating a direct association between periodontitis and NAFLD. These findings suggest that proper oral care, including periodontal treatment, may serve as an effective strategy for managing the comorbidity of periodontitis and NAFLD.

Periodontitis may contribute to the onset and progression of NAFLD by inducing oxidative stress and hepatocyte apoptosis. Evidence suggests that periodontitis triggers a systemic oxidative stress response, including in the liver (50). Consistently, our study found increased ROS levels in the livers of periodontitis-induced rats, along with reduced antioxidant biomarkers SOD and GSH. These findings confirm that periodontitis induces oxidative stress in hepatic tissue. Oxidative stress is recognized as a key driver of NAFLD progression. ROS activation of the NF-κB signaling pathway plays a crucial role in liver inflammation (51), and inhibition of NF-κB activation has been shown to reduce pro-inflammatory cytokine secretion (TNF-α and IL-6), thereby alleviating NAFLD (52). In agreement with previous studies, our results demonstrated upregulation of the MAPK and NF-κB pathways in the livers of periodontitis-induced rats. Additionally, oxidative stress promotes lipid peroxidation, exacerbating hepatic inflammation and fibrosis (53). Our data further support this, as periodontitis-induced rats exhibited significantly increased hepatic MDA levels. More importantly, proteomic analysis revealed substantial alterations in lipid metabolism-associated proteins in the livers of periodontitis-induced rats. These findings suggest that oxidative stress caused by periodontitis may disrupt lipid metabolism at the molecular level, leading to abnormal hepatic lipid accumulation and further accelerating NAFLD progression. While periodontitis-induced oxidative stress has been implicated in various systemic diseases (54), its role in lipid metabolism dysregulation within NAFLD remains underexplored. Our findings provide further evidence that oxidative stress-driven lipid metabolism disruption plays a critical role in the pathogenesis of NAFLD in the context of poor periodontal health.

Proteomic analysis of liver tissues from periodontitis-induced rats revealed that differentially expressed proteins were closely associated with fatty acid metabolism, particularly lipid metabolic processes such as fatty acid biosynthesis and degradation. Dysregulated hepatic fatty acid metabolism is a key driver of NAFLD onset and progression (55). Our findings, combined with previous research, highlight the role of periodontitis in promoting NAFLD through disruptions in hepatic fatty acid metabolism. Given the pathogenic significance of fatty acid metabolism dysregulation in NAFLD, targeting key proteins involved in this process may represent a promising strategy for managing NAFLD in the context of periodontitis.

In this study, we identified 56 differentially expressed LRPs and constructed a PPI network using STRING and Cytoscape software. By applying the cytoHubba plugin with nine algorithms, we identified the top ten hub proteins: Idh1, Dbt, Acox1, Pabpc1, Acaa2, Hadha, Egfr, Dlst, Eif3c, and VCP. DBT, a core component of the BCKD enzyme complex, plays a role in branched-chain amino acid (BCAA) catabolism and regulates lipogenesis. It may influence obesity and insulin resistance by suppressing fat synthesis (56, 57). Studies suggest that periodontitis elevates serum BCAA levels in mice, contributing to insulin resistance and NAFLD progression (58). In our study, DBT expression was downregulated in the livers of periodontitis-induced rats, potentially offering new mechanistic insights and therapeutic targets. ACOX1 is a key regulator of NAFLD development, responsible for peroxisomal β-oxidation of very long-chain fatty acids, under the control of PPARα (59, 60). In the liver, ACOX1 inhibits apoptosis and lipid accumulation (61), while periodontopathogens such as P. gingivalis can suppress ACOX1 expression, potentially exacerbating visceral fat accumulation and hepatic lipid dysregulation (62). ACAA2, the final enzyme in fatty acid β-oxidation, is stabilized by CAND1 through the Cullin1/FBXO42 pathway (63). Its expression significantly affects hepatic lipid metabolism and steatosis in NAFLD, though its role in periodontitis remains unexplored (64). HADHA, a key mitochondrial enzyme in long-chain fatty acid β-oxidation, is critical for lipid metabolism, and its downregulation leads to lipid accumulation and metabolic dysfunction (60, 65–67). Periodontitis may contribute to HADHA suppression through insulin resistance-induced glucagon elevation or oral-gut-liver axis infection, activating lysosomal degradation pathways and impairing fatty acid oxidation (68). Based on these findings, DBT, ACOX1, ACAA2 and HADHA emerge as potential key targets linking periodontitis to NAFLD. Further investigation into their regulatory mechanisms in periodontitis-associated NAFLD may provide novel strategies for mitigating lipid metabolism disorders and disease progression.

This study has several limitations. Firstly, due to the sample availability, we were unable to specifically examine the differential expression of critical inflammatory cytokines such as interleukins and TNFα, which play pivotal roles in NAFLD pathology. Furthermore, insulin resistance, a crucial metabolic marker associated with NAFLD progression, and high-sensitivity C-reactive protein (Hs-CRP), an essential indicator of systemic inflammation, were not assessed. These factors are vital for fully understanding the systemic inflammatory and metabolic interactions between periodontitis and NAFLD. Future studies should address these limitations by incorporating comprehensive evaluations of these inflammatory and metabolic biomarkers to provide deeper insights into the mechanisms connecting periodontal disease and NAFLD progression.

## 5 Conclusion

Our findings establish a direct link between periodontitis and NAFLD. Mechanistically, periodontitis induces hepatic oxidative stress and activates the MAPK and NF-κB signaling pathways, leading to disruptions in lipid metabolism through altered expression of key lipid-regulating proteins. This results in abnormal lipid accumulation, hepatocellular injury, and the progression of NAFLD. These results suggest that targeting periodontitis-induced oxidative stress and lipid metabolism dysregulation may enhance the precision of NAFLD treatment. Furthermore, the downregulation of DBT, ACOX1, ACAA2, and HADHA in the liver provides valuable insights for developing therapeutic strategies for periodontitis-associated NAFLD.

## Data Availability

The mass spectrometry proteomics data have been deposited to the ProteomeXchange Consortium (https://proteomecentral.proteomexchange.org) via the iProX partner repository (69, 70) with the dataset identifier PXD067109.
